# Interplay between Theory and Photophysical Characterization in Symmetric *α-Substituted* Thienyl BODIPY Molecule

**DOI:** 10.3390/molecules29112625

**Published:** 2024-06-03

**Authors:** Tersilla Virgili, Lucia Ganzer, Benedetta Maria Squeo, Arrigo Calzolari, Mariacecilia Pasini

**Affiliations:** 1 Istituto di Fotonica e Nanotecnologia—CNR, IFN—Piazza Leonardo da Vinci 32, 20133 Milano, Italy; lucia.ganzer@polimi.it; 2 Istituto di Scienze e Tecnologie Chimiche “Giulio Natta”—SCITEC—CNR, via Corti, 20132 Milano, Italy; squeo@scitec.cnr.it; 3 Istituto Nanoscienze—NANO—CNR, via Campi 213A, 41125 Modena, Italy; arrigo.calzolari@nano.cnr.it

**Keywords:** BODIPY, ultrafast spectroscopy, amplified spontaneous emission, density functional calculation

## Abstract

4,4-difluoro-4-bora-3a,4a-diaza-s-indacene (BODIPY)-based molecules have emerged as interesting materials for optoelectronic applications due to the possibility to easily fine-tune their photophysical and optical properties, dominated by two main absorption bands in the visible range. However, no studies have been reported on the nature of these spectral features. By means of ultrafast spectroscopy, we detect intramolecular energy transfer in a spin-coated film of di-thieno-phenyl BODIPY (DTPBDP) dispersed in a polystyrene matrix after pumping the high-energy absorption band. The same effect is not present upon pumping the lowest-energy band, which instead allows the achievement of efficient amplified spontaneous emission. Density functional calculations indicate the different nature of the two main absorption bands, explaining their different photophysical behavior.

## 1. Introduction

The rapid advancement of new technologies, ranging from nanomedicine and personalized medicine to stimuli-responsive smart materials [1] and organic electronics [2], presents unprecedented opportunities for progress across various fields and for addressing pressing social needs. Unfortunately, so far, the best-performing materials include rare elements such as lanthanide-based metal complexes [3,4] that do not meet the sustainability requirements crucial for modern society. In this context, organic dyes offer significant promise as their production does not depend on the extraction of rare earth elements, which are non-renewable resources. Furthermore, organic dyes provide a more accessible and environmentally friendly option for fulfilling the growing demand for high-performance materials.

Nonetheless, several challenges have to be overcome, including the development of organic dyes capable of absorbing and emitting light in the red and near-infrared regions. One of the most promising materials in this regard is 4,4-difluoro-4-bora-3a,4a-diaza-s-indacene (BODIPY) [5]. This fluorescent organic chromophore [6] has garnered significant attention across various fields due to its versatile properties and wide range of applications [7,8]. Its unique chemical structure is characterized by a boron–dipyrrin core, which offers several advantages, including strong absorption and emission in the visible-to-red region of the spectrum, high-fluorescence quantum yields, excellent stability and tunable optical properties through structural modifications [9,10,11].

**Scheme 1:**
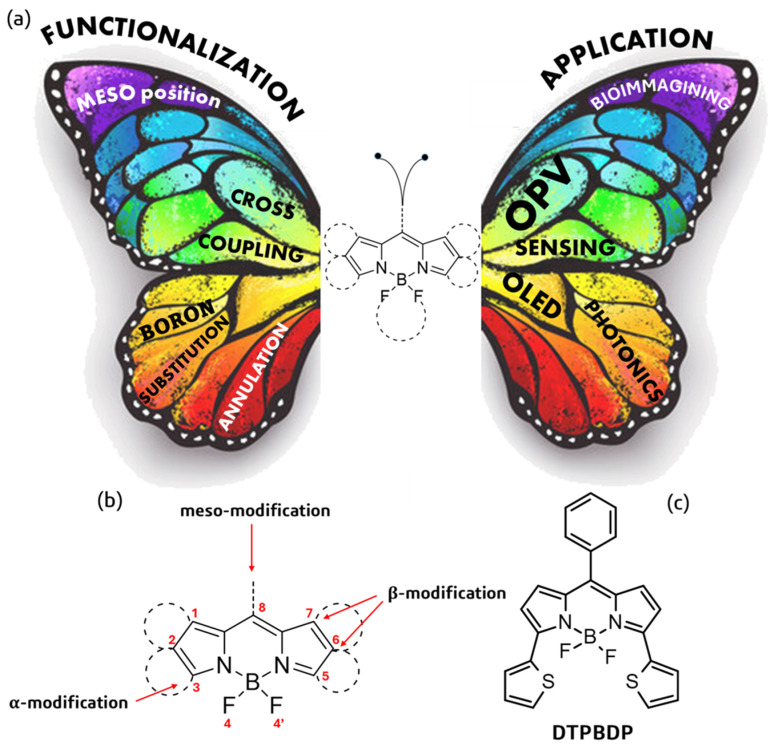
(**a**) Schematic representation of possible functionalization on BODIPY’s core and possible application of BODIPY derivatives; (**b**) representation of the eight possible functionalization sites of BODIPY; (**c**) chemical structure of the BODIPY derivative di-thieno-phenyl BODIPY (DTPBDP).

Over the years, several synthetic strategies have been employed to fine-tune the optoelectronic properties of BODIPY [12]. Depending on the position and type of substituents, various parameters, such as the delocalization of π-electrons in the molecule and the supramolecular organization and morphology of solid thin films, can be modified. In this regard, the BODIPY core has eight reactive positions (See Scheme 1) [13] that can be used to modulate its optical properties [14,15]: two α-positions; four β-positions, which have the greatest influence on electronic delocalization; and two other positions, meso and Boron, which have a strong impact on the steric hindrance of the molecule and consequently on the thin-film properties. The substitution with electron-rich thienyl moieties is one of the main strategies used to downshift the energy of the absorption and emission of the molecules [16], and it has also been successfully reported for BODIPY-based dyes [17,18,19,20]. These modifications involve introducing thiophene-containing moieties to the BODIPY core [21], facilitating the extension of conjugation and emission spectra. Additionally, thienyl-annulated BODIPYs have shown enhanced singlet-triplet conversion quantum yields, further enhancing their utility in various applications [22].

The study of thienyl-substituted BODIPY and its derivatives is crucial for advancing fields such as chemical sensing, biomedical analysis and labeling, light-harvesting systems, dye-sensitized solar cells and non-linear optical materials. Understanding the impact of thienyl modifications on the structure–property relationships of BODIPY compounds can lead to the design of next-generation fluorescent probes with improved performance and broader applicability in biomedical and optoelectronic applications. (Scheme 1) For this reason, we have synthetized a symmetric thienyl-substituted BODIPY in the two α-positions (DTPBDP; see Scheme 1).

The study of thin films based on BODIPY derivatives holds significant importance in the field of photonics [23]. These materials exhibit promising characteristics, such as a high photoluminescence quantum yield and ease of functionalization, making them ideal candidates for active materials in photonics applications. While BODIPY derivatives have shown exceptional performance in diluted solutions as lasing media, their application in the solid state has been less explored due to challenges such as a lower quantum yield and susceptibility to the composition and structure of polymer matrices [24,25].

In this paper, we studied the nature of the two main absorption bands of a thin film of a BODIPY-based molecule, the dithieno-phenyl BODIPY (DTPBDP see Scheme 1c) dispersed in an inert matrix. Using ultrafast spectroscopy, we found that pumping the high-energy band induces slow intramolecular energy transfer (IET) that prevents efficient amplified spontaneous emission (ASE) from the molecule, present instead after excitation of the low-energy absorption band. Density functional calculations confirmed the different exciton localization upon excitation at high or lower energy. Our results pave the way to a more complete understanding of the photophysics of those molecules.

## 2. Results and Discussion

DTPBDP (Scheme 1c) was prepared according to literature [26,27] with a slightly modified procedure (see Supporting Information (SI)). Differently from previous reported papers, the bromination of the phenyl dipyrromethane (M1) was followed by oxidation with 2,3-Dichloro-5,6-dicyano-1,4-benzoquinone (DDQ) without further purification, and the subsequent closure of the cycle with boron trifluoride diethyl etherate, to obtain dibromo-BODIPY (M2); see Supporting Information (SI). The α-substituted thienyl BODIPY (DTPBDP) was synthesized by Stille Coupling as reported in the literature [28].

Aggregation-caused quenching (ACQ) and the reabsorption of fluorescence derived from high planarity and a small BODIPY Stokes shift may cause a decrease in the fluorescence quantum yield. In order to avoid these problems, the dye was dispersed in a polystyrene matrix as previously reported in the literature [29,30]. A non-polar matrix was chosen to avoid solvatochromism [31] in BODIPY-based molecules, which could complicate spectroscopic analysis. Indeed, various contributions lead to optical red shifts, such as solid-state planarization, aggregate formation and, if present, the polarity effect of the matrix [24,28]. The resulting films are homogeneous with good dispersion of the dye. 

**Figure 1:**
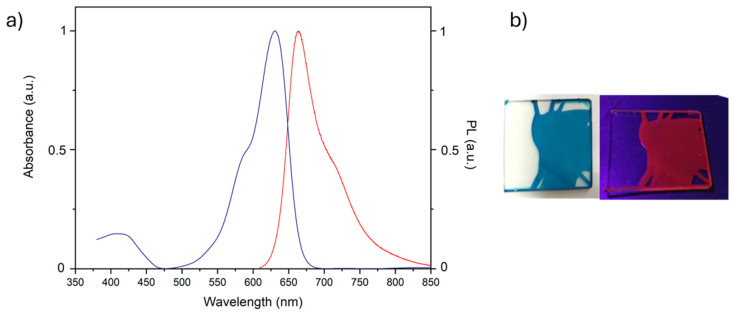
(**a**) Absorption (blue line) and photoluminescence (red line PL) spectra from spin-coated film; (**b**) picture under ambient light (left) and under 366 nm light (right) of spin-coated film.

Figure 1 shows the absorption and the photoluminescence (PL) spectra of the spin-coated film (10% dye in polystyrene (PS)—see Materials and Methods for details). The absorption spectrum presents two bands in the visible range: one at high energy at 400 nm, and the second at 631 nm with a vibronic replica at 585 nm. These bands are attributed to S_0_-S_1_ (631 nm) and at S_0_-S_n_ (400 nm) transitions, respectively. The emission spectrum presents a main peak at 665 nm with a vibronic replica at 715 nm.

**Figure 2:**
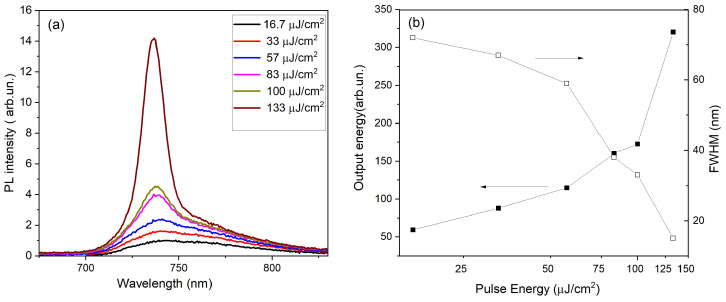
(**a**) Photoluminescence spectra with lateral detection after excitation at 645 nm at different pump fluences. (**b**) Output PL energy and linewidth of the main peak at different pump fluences.

Amplified spontaneous emission measurements (ASE) [32,33] were performed on this sample by pump excitation at 400 nm (S_0_–S_n_ transition) and at 645 nm (S_0_–S_1_). Under 400 nm pump excitation, no ASE was detected, while at 645 nm, narrowing of the emission is present. Figure 2a reports the PL spectra after 645 nm pump excitation as a function of the excitation energy density. 

Even at low excitation density, the spectra present a red shift in the spontaneous emission peak (from 660 to 740 nm), ascribable to strong self-absorption along the thin film. As the excitation density increases above 50 μJ/cm^2^, a clear narrow band appears at about 735 nm, progressively dominating the spectra. This feature is typical of ASE, with an estimated threshold of about 55 μJ/cm^2^, corresponding to the excitation density at which the narrow ASE peak rises up, and its linewidth starts to decrease (see Figure 2b). The sharp ASE peak at 735 nm corresponds to the 0–1 vibronic emission peak (Figure 1). No degradation is observed during the measurements. 

To understand the different behavior after excitation at high and low energy, we performed ultrafast spectroscopy excitation at 400 and at 650 nm. 

**Figure 3:**
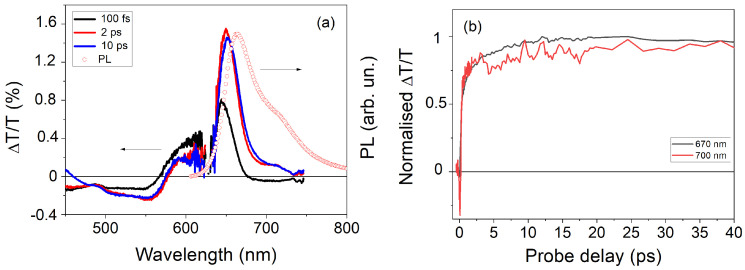
Pump excitation at 400 nm. (**a**) Transient transmission spectra at different probe delays (solid lines). Photoluminescence spectrum is represented by open circles. (**b**) Time decays at 670 nm and at 700 nm.

Figure 3a shows the transient transmission spectra at different probe delays in comparison with the PL spectrum after excitation at 400 nm. The spectra consist of a positive band from 570 nm to 740 nm, and a negative one from 450 to 570 nm. The positive band features three peaks at 585 nm, 650 nm and 710 nm.

The first peak represents the contribution of ground-state bleaching (GSB), while the peaks at 650 nm and 715 nm represent the stimulated emission. Figure 3b shows that the stimulated emission is not instantaneous and presents a slow growing time of around 10 ps. 

**Figure 4:**
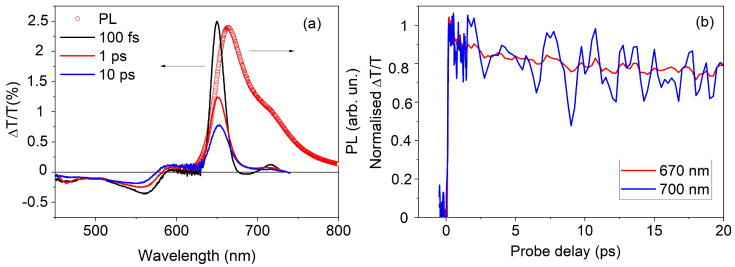
Pump excitation at 650 nm (**a**) Transient transmission spectra at different probe delays (solid lines). Photoluminescence spectrum is represented by open circles. (**b**) Time decays at 670 nm and at 700 nm.

Figure 4a shows the transient transmission spectra at different probe delays in comparison with the PL spectrum after excitation at 650 nm. In this case, the pump–probe spectra at different probe delays are similar to the ones after pump excitation at 400 nm, while the dynamics are different. The signals at 670 nm and 700 nm are instantaneously created and not delayed as with different pump excitation values.

**Figure 5:**
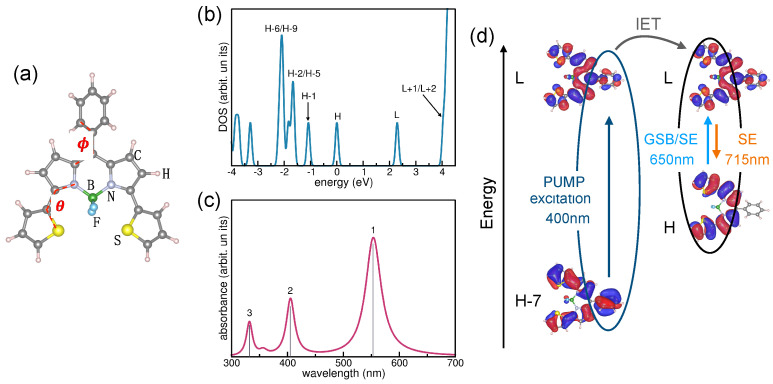
(**a**) Ground-state geometry (GGA-PBE) of the DTPBDP molecule. Black labels denote the chemical species (C, N, B, F, S, H). Red dashed lines indicate the dihedral angles between the phenyl ring and the pyridine core (*ϕ*) and the pyridine core and thiophene rings (*θ*), respectively. (**b**) Electronic density of states (DOS) of minimum energy of the DTPBDP molecule (B3LYP). The zero-energy reference is set to the HOMO (H) state of the molecule. (**c**) Simulated absorption spectrum (B3LYP). Labels **1**–**3** indicate the main low-energy peaks. (**d**) Pictorial sketch of the excitation (left) and de-excitation (right) processes with intramolecular energy transfer (IET).

To gain a microscopic understanding of this behavior, calculations were performed within the density functional theory (DFT) framework (see Materials and Methods). We considered several initial atomic structures that differed in the out-of-plane rotation of the phenyl and thiophene rings and of the diflouroboryl groups. All relaxations were carried out at a GGA-PBE level of accuracy. After atomic relaxation, the minimum-energy geometry is highly nonplanar (Figure 5a), with the phenyl and the thiophene rings rotated by ϕ=53° and θ=27°, respectively, with respect to the pyridine core, while the fluorine atoms settle almost perpendicular to the central C_3_N_2_B ring. The rotation of the lateral groups affects the overall π-conjugation across the molecule, as described below. 

The electronic and optical properties of the DTPBDP molecule were calculated at the B3LYP level, by keeping the atoms fixed at the minimum-energy GGA-PBE geometry. The electronic density of states (DOS) is shown in Figure 5b. All frontier orbitals have a π-like character, whose spatial distribution varies in the different states. In the highest occupied molecular orbital (HOMO—H in Figure 5b), the charge density is delocalized along the C3N2 units and the lateral thiophene tails, with zero contributions from the central phenyl ring. A similar character is observed for the multiplet of states H-2/H-5. On the contrary, most of the closest orbitals, including the lowest unoccupied molecular orbital (LUMO—L in Figure 5), L + 1, L + 2, as well as H-1 and the multiplet H-6-H-9, have a uniform charge density distribution which spans the entire molecule, including the phenyl head. The comparison with the results obtained at the GGA-PBE level confirms that—except the expected enlargement HOMO-LUMO gap—no further remarkable modifications appear either in the energy distribution (i.e., DOS) or in the symmetry of the single-particle molecular orbitals (see Supporting Information, SI). The simulated B3LYP absorption spectrum is shown in Figure 5b. The low-energy part of the spectrum is dominated by three main peaks (labeled **1**–**3** in Figure 5c). Apart from a small and systematic shift due to the XC limitations of TDDFT, the spectrum effectively reproduces the features of the experimental measurements, with a main sharp peak at 554 nm (peak **1**) and a broad lower intensity band between 330 and 410 nm which encompasses peaks **2** and **3** and a small shoulder in between. The analysis of the spectral features in terms of single-particle occupied–unoccupied electronic transitions (Table 1) indicates two different kinds of excitations. Peak **1** is mainly associated with the H→L transition with a minor contribution from the H-5→L transition. Both H and H-5 orbitals do not have charge density distribution across the phenyl group, at odds with the final LUMO state. Peaks **2** and **3** instead involve transitions between states that are fully delocalized on the entire molecules (see Table 1). As illustrated in Figure 5d, this goes along with the different behavior observed in the pump–probe measurements discussed above: the pump at 400 nm excites the transition between fully π-conjugated states; then, through an intramolecular energy transfer between the phenyl head and the remaining π-conjugated part of the molecule, the de-excitation H→L occurs. 

## 3. Materials and Methods

### 3.1. Film Preparation

The films were prepared on quartz glass by spin-coating a solution (100 mg/mL) at 1500 rpm for 60 s in toluene of DTPBDP (10% *w*/*w*) and PS (Aldrich, Darmstadt, Germany, Mn 140000, Mw 230000).

### 3.2. Ultrafast Spectroscopy

The ultrafast spectroscopy setup was fed by a 150 fs, 2 kHz repetition rate Ti:sapphire system (Libra, Coherent, Santa Clara, CA, USA) with a central wavelength of 800 nm. Transient-absorption measurements were performed by pumping at 400 nm with the second harmonic of the laser output, generated with a 1 mm, Type I β-barium borate crystal, and at 640 nm using a non-collinar optical parametric amplifier (NOPA). The probe pulses, with a spectrum spanning from 450 to 750 nm, were obtained by white-light generation in a 3 mm thick sapphire crystal. The measurements were performed in transmission, and the probe spectrum was detected using a SP2150 Acton, Princeton Instruments spectrometer. The pump beam was modulated by a mechanical chopper at a 1 kHz frequency, and the differential transmission (ΔT/T) spectrum of the probe was measured as a function of probe wavelength and pump–probe delay. The polarization between pump and probe beams was set at magic angle (54.7°).

### 3.3. ASE Measurements with Femtosecond Excitation

ASE was obtained by pumping the film at 400 nm and at 640 nm with rectangular stripe spot excitation with a length of 1.05 mm and a width of 0.140 mm (1.5 × 10^−3^ cm^2^). The emitted spectrum was collected at the edge of the film with the help of a spectrograph (Princeton Instruments SP2150, Acton, MA, USA, 300 gr/nm) coupled with a CCD camera (Princeton Instruments pixis 256). The spectral resolution was 0.5 nm. All the measurements were performed in air.

### 3.4. Theory and Simulations

Simulations were performed using (time-dependent) density functional theory ((TD) DFT) [34] with GGA-PBE [35], and hybrid B3LYP [36,37] exchange-correlation (XC) functionals. Optimized norm-conserving Vanderbilt pseudopotentials were used to treat the ionic potentials [38,39]. Single-particle Khon–Sham orbitals (charge density) were expanded in plane waves up to a kinetic-energy cutoff of 48.0 Ry (192 Ry). DTPBDP molecules were simulated in a (30.3 × 32.0 × 20.0) Å^3^ cell; this assures a minimum of ~2 nm distance between adjacent replicas. Γ-point only was used for charge density integration in the reciprocal space. Ground-state calculations and structural optimizations were performed with the pw.x code included in the Quantum ESPRESSO (QE) distribution [40,41]. The convergence thresholds for the geometrical optimizations were set to 0.03 eV/Å. Excited states were studied within TD density functional perturbation theory using the turboTDDFT components of QE [42], which includes a Davidson-like approach [43] to the solution of Casida’s equation for the analysis of the lowest-energy optical transitions. The transition dipole was calculated in real space, and the residue convergence threshold in the solution of the Davidson problem was set to 10^−4^. A Lorentzian broadening of σ = 0.01 Ry was used to simulate the absorption spectrum.

## 4. Conclusions

In this paper, we studied the nature of the two main absorption bands of a thin film of a DTPBDP molecule dispersed in an inert matrix. The absorption spectrum is mainly characterized by two absorption bands at 400 nm and at 631 nm. Using ultrafast spectroscopy, we found that pumping the high-energy band induces a slow intramolecular energy transfer, which prevents efficient amplified spontaneous emission (ASE) from the molecule, which instead occurs after excitation of the low-energy absorption band. Density functional calculations confirmed the different exciton localization upon excitation at high or lower energy. Our findings pave the way for establishing a deeper understanding of the photophysics of this molecule class, thereby contributing to the enhancement of material design for more high-performance materials tailored to specific applications.

## Figures and Tables

**Table 1:** LYP XC functional. Peak enumeration follows Figure 5b. Main transitions are marked in bold. Values in parentheses indicate the percentage contribution of the most relevant single-particle transitions.

Peak	Wavelength	Transitions
**1**	554 nm	**H→L** (90%), H-5→L (5%)
**2**	406 nm	**H-1→L** (70%), H→L + 2 (16%)
**3**	331 nm	**H-7→L** (65%), H-6→L (20%), H-8→L (4%)

## Data Availability

Data are contained within the article and Supplementary Materials.

## Supplementary Materials

The following supporting information can be downloaded at: https://www.mdpi.com/article/10.3390/molecules29112625/s1, Figure S1. Comparison between electronic density of states (DOS) of BODYPI molecule calculated at (a) the GGA-PBE level, and (b) by using hybrid B3LYP exchange-correlation functional (bottom panel); Figure S2. Comparison between absorption spectrum of BODYPI molecule calculated at (a) the GGA-PBE level, and (b) by using hybrid B3LYP exchange-correlation functional (bottom panel).
