# Supplementary material for: Interplay between Theory and Photophysical Characterization in Symmetric α-Substituted Thienyl BODIPY Molecule
Source: Molecules. 2024 Jun 3;29(11):2625. doi: 10.3390/molecules29112625 (PMC11173456; doi:10.3390/molecules29112625)
Supplement: Supplementary file 1 [file molecules-29-02625-s001.zip › molecules-2985990-supplementary.pdf]

Supporting Information

Interplay between Theory and Photophysical Characterization in Symmetric *α-Substituted* Thienyl BODIPY Molecule

Tersilla Virgili, Lucia Ganzer, Benedetta Squeo, Arrigo Calzolari, Mariacecilia Pasini

General Information for synthesis. All reagents were purchased from commercial source and used without further purification. All solvents have been distilled prior to use. All reaction were carried out in inert atmosphere. The ^{1}H NMR spectra) were recorded with a Bruker ARX 400 MHz spectrometer (Bruker, Karlsruhe, Germany). Gas-phase mass determination was carried out using the Agilent Technologies 7890A GC System (Santa Clara, CA, USA) coupled with an Agilent Technologies 5975C VL MSD (Santa Clara, CA, USA) with a triple-axis mass detector.

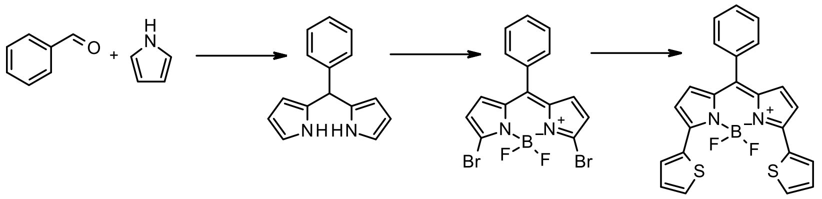

M1 M2 DTPBDP

*Synthesis of monomer **M1***. Benzaldehyde (1g, 9,4 mmol) was dissolved in excess of pyrrole (25,2 g, 40 eq). The resulting mixture was degassed for 30 min by nitrogen and then 100 µl of trifluoracetic acid were added. The reaction was stirred at room temperature for an hour and the crude product was diluted with dichloromethane and washed three times with sodium hydroxide (NaOH) aq. 0.1 N and dried on magnesium sulphate. The solvent was removed under reduced pressure. The product was purified by column chromatography on silica gel using hexane/ethyl acetate 9:1 as eluent. The pure product was obtained as a brown powder with a yield of 66% (1.360 g). Purity controlled with GC-MS (m/z = 222.1).

*Synthesis of monomer **M2***. Monomer **M1** (600 mg, 2,7mmol) was dissolved in dry THF and cooled down to −78 °C under nitrogen. N-Bromosuccinimide (961 mg, 5,4 mmol) was added portionwise in three time over 10 min. After one hour, the reaction mixture was controlled by GC/MS, showing only the dibromoderivative and a slight amount of monobrominanted compound. The reaction mixture was then warmed to room temperature. A solution of dry THF (15 ml) and DDQ (612 mg, 2,7 mmol) was added dropwise to the reaction mixture in 10 minutes. After 10 minutes, the solvent was removed and the crude product was quickly purified on silica gel using dichloromethane as eluent.

The compound was then redissolved in dry toluene (35 ml), DIPEA (1,640g, 12.69 mmol) was added and the mixture stirred at room temperature for 90 min. Finally the boron trifluoride diethyl etherate (5.7 mL) was added and the mixture was stirred at 80 °C for 2 h and then cooled at room temperature. The crude product was washed with water and dried on magnesium sulphate. The solvent was removed under reduced pressure and the product was purify by silica gel chromatography using a mixture of hexane/ ethyl acetate 8:2 as eluent. The pure product was obtained as shine dark violet crystalline powder with a yield of 49% (561 mg). ^{1}H NMR (400 MHz, $CDCl_3$): δ 7.50-7.45(m, 5H) 6.84(d, J = 4.3 Hz, 2H), 6.54 (d, J = 4.3 Hz, 2H).

*Synthesis of **DTPBDP**.* Monomer **M2** (100mg, 0,24mmol), tetrakis(triphenylphosphine) palladium(0) $Pd(PPh_3)_4$ and stannyl thiophene were refluxed in dry toluene under nitrogen and under stirring. After 24 hours the reaction mixture was diluted in dichloromethane and filtered on celite pad. The solvent was removed under reduced pressure and the crude product purified by silica gel chromatography using hexane/dichloromethane 7:3 as eluent. The pure product was obtained as shine dark blue crystalline powder with a yield of ^{1}H NMR (600 MHz, $CDCl_3$): δ 8.2(d, J=3.8 Hz, 2H) 7.55-7.50 (m, 5H) 7.48(d, J=4.3Hz, 2H) 7.20 (t, J=4.3 Hz, 2H) 6.81-6.78 (m, 4H).

2. Theoretical calculation

Comparison between electronic density of states (DOS) of BODIPY molecule calculated at the GGA-PBE level, and by using hybrid B3LYP exchange-correlation functional.

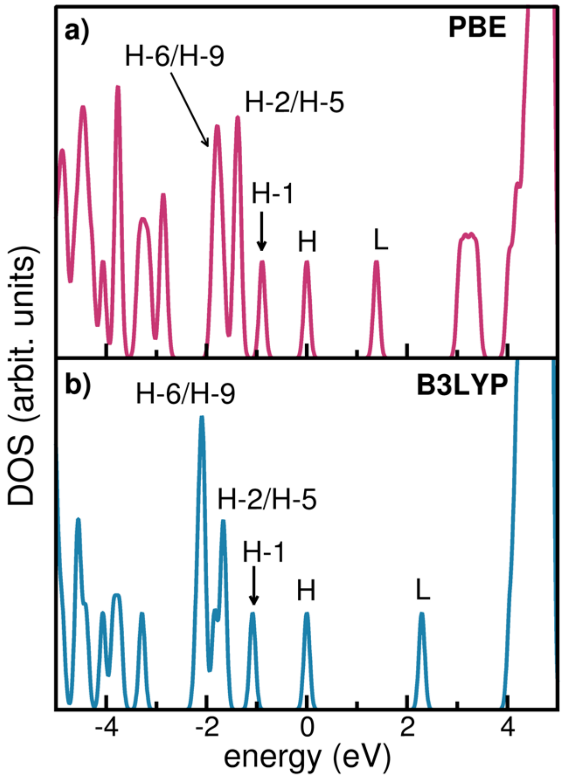

Figure S1. Comparison between electronic density of states (DOS) of BODIPY molecule calculated at (a) the GGA-PBE level, and (b) by using hybrid B3LYP exchange-correlation functional (bottom panel).

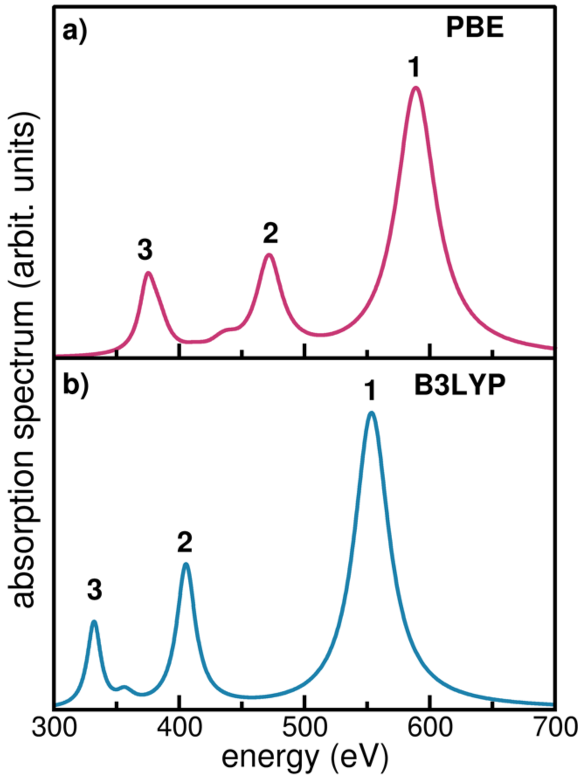

Figure S2. Comparison between absorption spectrum of BODIPY molecule calculated at (a) the GGA-PBE level, and (b) by using hybrid B3LYP exchange-correlation functional (bottom panel).
